# Good psychiatric management for borderline personality disorder: A qualitative study of its implementation in a supported employment team

**DOI:** 10.1371/journal.pone.0299514

**Published:** 2024-03-15

**Authors:** Noëllie Dunand, Philippe Golay, Charles Bonsack, Danièle Spagnoli, Valentino Pomini

**Affiliations:** 1 Institute of Psychology, Faculty of Social and Political Science, University of Lausanne, Lausanne, Switzerland; 2 Department of Psychiatry, Community Psychiatry Service, Lausanne University Hospital and University of Lausanne, Lausanne, Switzerland; University of Connecticut Health Center: UConn Health, UNITED STATES

## Abstract

**Introduction:**

People with borderline personality disorder have difficulties with work. The Individual Placement and Support model has shown its worldwide effectiveness in terms of vocational rehabilitation for individuals with psychiatric disorders. However, only a few recent studies have explored its results for people with personality disorders, and the findings were mitigated. Additionally, Individual Placement and Support job coaches reported difficulties in supporting this population. An evidence-based psychotherapeutic method, also applicable in a case management context, called Good Psychiatric Management for borderline personality disorder, could potentially overcome these obstacles. This study aimed to evaluate the initial integration of Good Psychiatric Management in Individual Placement and Support practice.

**Methods:**

Individual Placement and Support practitioners of Lausanne University Hospital, Switzerland, were trained in Good Psychiatric Management in January 2022. Five of them participated in a focus group to collect their impressions about the training, and six were interviewed 9 months later to assess the initial adoption of Good Psychiatric Management into their practice. Thematic analyses were conducted.

**Results:**

Job coaches were positive about this new tool. All of them found it useful and beneficial both for them and their patients. They were able to follow the main Good Psychiatric Management principles in their practice However, the findings also suggested some additional improvements in the implementation process.

**Conclusions:**

Integrating Good Psychiatric Management in Individual Placement and Support seems feasible, and the team who appreciated it adopted it. The method offers new perspectives in community support for people living with borderline personality disorder.

## Introduction

People with personality disorders (PD), especially of the borderline type, show work-related dysfunctions such as interpersonal difficulties, impulsivity [1], a low education level, work conflicts, which often result in losing a job on purpose [2], dismissals, demotion, and unemployment [3, 4]. Moreover, employers are critical towards staff members with relationships issues, difficulties in admitting their own mistakes, mood swings, and resistance to instructions, which PD patients often convey [5].

To help people with mental illness in their professional rehabilitation, the Individual Placement and Support (IPS) model [6] of supported employment has shown worldwide efficiency [7]. It consists of job coaches accompanying patients to work and helping them to maintain their job, adapting to their needs and preferences for as long as they wish. The goal is to rapidly access the competitive work market, defined as regular paid job available to everyone. IPS participation is open to anyone receiving treatment for a mental illness. Job coaches collaborate with healthcare teams and develop connections with the job market.

However, IPS model was conceptualised for people suffering from severe mental illnesses [8]. Those refer to disorders that persist over time and are prone to relapse and recurrence such as schizophrenia and chronic mood disorders [9]. Only few recent studies have explored IPS effectiveness for people with PD, the results of which were not conclusive. One of them showed no difference between traditional IPS clients and those with a PD but cited the small sample size and heterogeneity of the PD groups as possible explanations for this result. [10, 11]. To avoid the issue of heterogeneity, Dunand et al [12] studied the IPS population with PD divided into the DSM-5 clusters [13]. They notably found that people in clusters A and especially B had a lower rate of professional reintegration and a slower time to reach employment than people with a PD in cluster C or with disorders other than PD. Also, IPS principles consistency with guidelines for treating people with PD is questionable. The latter advocates more structure than IPS, which is time-unlimited and focuses on patients’ preferences. Furthermore, as professionals in other clinical settings, notably with medical, nursing and social work background [14], IPS job coaches report more difficulties in their practice when encountering people with this disorder. This shows the necessity for adapting IPS to PD patients.

An evidence-based therapeutic method, Good Psychiatric Management (GPM) for borderline personality disorder (BPD) [15] is currently being expanded. It is based on best practices recommended by the American Psychiatric Association [16] for the treatment of BPD. It consists in flexible guidelines of attitudes to adopt when facing patients with this condition. Its basic principles are to offer psychoeducation, not overreact, be cautious, value the relationship, convey that change is expected, foster accountability, maintain a focus on life outside of treatment and be pragmatic. A randomised-controlled trial showed that its effects and lasting characteristics on patients equalled those of dialectical behaviour therapy [17, 18]. These results should be interpreted with caution given that research on the topic is scarce. Patients who fail to respond should be advised to follow specialised treatments. Nevertheless, GPM is effective for most patients and covers basic training of mental health professionals. It is recommended not only in therapy contexts but also in case managers’ practices [19]. Therefore, it could be considered a potential additional feature that could be relatively easily integrated into the IPS programme, without underestimating the cost in time and resources of incorporating new elements into one’s practice. Evidence shows that a one-day training in this approach already increases practitioners’ comfort with and interest in working with BPD patients [20, 21]. Moreover, GPM and IPS share common values and practices, such as patients’ empowerment, focus on vocational integration, use of common sense, importance of setting goals and multimodality of treatment.

Therefore, this study aimed to assess through a qualitative study the initial integration of GPM for BPD into IPS practice. We hypothesised that this integration was successful in terms of feasibility, acceptability, appropriateness, adoption, and fidelity, as based on Proctor et al. Proctor et al [22] outcomes for implementation research.

## Methods

### Design

This study was conducted in RESSORT, a community network programme for supported employment from the Community Psychiatry Department of Lausanne University Hospital—including three centres: Lausanne, Prangins and Montagny-Près-Yverdon—and Nant Foundation in Montreux (Switzerland). IPS was implanted at RESSORT in 2009. A team that the founders of IPS in Montreal (Canada) supervised then trained a part of RESSORT’s team members on the model. RESSORT’s coordinator was trained as an IPS supervisor. Since then, the initial team members internally train co-workers with course material that the IPS founders validated. The specificity of RESSORT IPS team is that it is part of the hospital’s public services. The patients’ treatment team is not directly attached to the service. Instead, anyone suffering from a mental illness and being treated by a psychotherapist is allowed to join, and job coaches are in regular contact with them. In addition, Switzerland’s economic context, characterised by higher educational standards, fewer entry-level jobs, and difficulty laying off workers, influences the model as compared to the labour market in the United States, where IPS was created. As a result, this team has fair fidelity to the original model according to the IPS fidelity scale [23]. Due to the above-mentioned particularities, the items that received the lowest ratings included the contacts between IPS team and the treatment team, the rapid start-up of job searches, and the creation of links with the job market. IPS job coaches follow around 15 to 20 patients for a full-time position, of which approximately one third have a PD.

For this study, all 12 IPS job coaches from RESSORT were trained to use GPM for BPD. They received a half-day training, which took place online due to the COVID-19 pandemic-related measures in January 2022. Since then, they benefit from an ongoing monthly group supervision by an expert in the GPM approach. According to Proctor et al [22], implementation outcomes should include the service at different stages. Therefore, qualitative data were collected at two timepoints in RESSORT with IPS job coaches.

### Sample

The research team offered job coaches from RESSORT to participate voluntarily in this study. They were recruited through existing collaborations within our psychiatry service between the 20^th^ of January 2022 and 13^th^ of December 2022. All participants signed written informed consent forms. The Human Research Ethics Committee of the Canton Vaud approved the project (protocol # 2021–01362).

We conducted a focus group with five job coaches, who came forward to take part in the study, including four women and one man. Two of them were nurses, one an occupational therapist, one a social educator, and one a psychologist. The average age was 43.8 years (range: 30–60), number of years of experience as a job coach was 3.1 (range: 0–7), and number of years of experience in psychiatry prior to their current job was 13.4 (range: 3–25). Their average work rate as IPS job coaches was 70% (range: 20–100%).

Regarding number of individual interviews, we aimed at reaching saturation, usually emerging between six and 12 interviews [24]. In total, we interviewed six female job coaches, two of whom had participated in the focus group. Two participants were psychologists, two social workers, one an occupational therapist, and one a social educator. They were selected as they showed an interest in participating. The average age was 38.2 years (range: 30–43), number of years of experience as a job coach was 3.5 (range: 1–8), and number of years of experience in psychiatry prior to their current job was 5.3 (range: 3–11). IPS job coaches’ work on average at 76.6% (range: 50–100%).

#### Procedure

A focus group and individual interviews of around 1 hour were conducted with the job coaches in their office building to collect participants’ opinions about the integration of the GPM training for their practice. They were audio-recorded, transcribed, and anonymised.

The first author led the focus group 6 weeks after the GPM training to obtain their initial thoughts as a group about the relevance of the collective training soon after attending it. Their opinions about the integration of GPM in their practice, its benefits and limitations, its compatibility with IPS, how they thought it would modify their practice, and what would be their needs for an optimal implementation were asked through semistructured questions.

The first author and a graduate psychology student conducted each semistructured interview around 9 months after the GPM training to assess individually the change in their practice, and more specifically, the implementation’s relevance, acceptability, feasibility, adoption, and fidelity [22]. The topic guide concerned change in their practice that potentially emerged following the training, its benefits, limitations, which principles they did or did not adopt, to which extent they found GPM compatible with IPS, and the relevance of implementing this training on a larger scale. During both the interviews and the focus group, job coaches were presented with GPM and IPS principles on a sheet to help them discuss their relevance and compatibility.

### Data analyses

A thematic analysis [25] was performed over the focus group and individual interviews’ content. It consisted of finding sense by reorganising data according to emerging topics. Each interview portion could be coded more than once. Parts of the interview that did not answer the research questions were not coded. The first author coded the focus group and the individual interviews of patients, and, to increase interpretation objectivity, a graduate psychology student helped with coding the entire data set of coaches’ individual interviews. Content was segmented into meaningful features, which were then collated and gathered into themes and codes. When analysing the sixth interview, because no more new code was generated and the entire data set converged into identical themes, saturation was assumed to have been reached and sampling stopped. Additionally, the frequency of participants mentioning each theme and code helped determine the section’s representativeness for the sample [26]. A fragment was considered “general” when all the participants mentioned it, “typical” when five or four participants mentioned it, “variant” when three or two participants mentioned it, and “rare” if only one participant mentioned it.

### Researchers’ reflexivity

We adopted a reflexive attitude during the research process to reduce possible biases in qualitative studies of the coconstruction of meaning, with the aim of preserving the authenticity of participants’ discourse. The first author is trained as a psychologist and works regularly with the job coaches. However, she is not involved in the patients’ treatment within or outside the present research context. Analyses were discussed with other team members, including senior research and clinical psychologists, and a senior psychiatrist, as well as during interdisciplinary meetings comprised of clinicians, researchers, peer practitioners, and patients. The codes and themes were reviewed together in the light of some interview quotes. The researchers’ assumptions and analyses were presented, and other team members then examined whether other interpretations of the same data could be plausible. The conceptualisation of the project and the interpretation of results were discussed several times. What emerged from these discussions was taken into account in the final work.

## Results

The implementation assessment results are displayed in two parts: (a) according to the team soon after the GPM training, and (b) according to the team 9 months after training.

In each part, themes follow elements of success, satisfaction, dissatisfaction, and prospects of improvement. Themes and subthemes related to positive attitudes were generally more developed than the ones regarding negative aspects.

*Part 1*: *Service implementation outcomes soon after the GPM training*

Table 1 displays results of the discourse analysis of the focus group that took place with job coaches a few weeks after participating in the training. Four themes have been highlighted, all general in terms of representativeness of the sample.

**Table 1:** Analysis of individual placement and support job coaches’ discourse regarding the integration of good psychiatric management few weeks after training.

Themes	Codes
Positive impact of the GPM training in the context of IPS (*N* = 5)	Useful tool (*N* = 5) Relevant reminder to take care of the beginning of the intervention (*N* = 4) Interesting principles (*N* = 4) Elucidation of spontaneously applied principles (*N* = 3) Compatibility with RESSORT (*N* = 3) Structure (*N* = 3) Expected benefits for patients (*N* = 3) Practice assessment allowance (*N* = 3) No detrimental aspect (*N* = 2) Adaptation of IPS to BPD patients allowance (*N* = 2) Solution to the difficulties (*N* = 2)
Lacks in the training (*N* = 5)	Need for contacting patients’ former employers not addressed (*N* = 4) Motivation problems not addressed (*N* = 4) Problems in setting an intervention goal not addressed (*N* = 3) Work capacity-related problems not addressed (*N* = 3) Full adaptation of IPS to BPD patients not allowed (*N* = 2) Swiss economy-related problems not addressed (*N* = 2)
Conditions to apply GPM (*N* = 5)	Care network collaboration necessary (*N* = 5) BPD diagnosis necessary (*N* = 4) Need for discussion times after the training to integrate GPM (*N* = 4) Need for financial resources for optimal implementation (*N* = 2)
Negative aspects of the GPM training in the context of IPS (*N* = 5)	Incompatibility with the role of job coach (*N* = 4) Additional workload (*N* = 3) Difficulty in automatically applying GPM principles in practice (*N* = 3) Incompatibility with certain IPS principles (*N* = 2)

*Note*. IPS = Individual Placement and Support; GPM = Good Psychiatric Management; BPD = borderline personality disorder.

The first theme, being the one containing the most codes, gathers the positive points of the training according to the job coaches. This includes an interest in the tool and principles presented; the recognition of aspects already present in their practice, hence, GPM compatibility with the service; its advantages for both users and providers; and its relevance for assessing the intervention. A participant described the benefits of the training as being a reminder of good practices to use with patients with BPD.

We must be vigilant when we enter the relationship because we know that this is what is very important, and to frame and perhaps even anticipate the intervention and put in place (…) an action plan that is a little more defined, formalised, personalised, in order to anticipate the moments when it will be more difficult. So, for me, it’s true that it was really a little alarm bell that told me to be careful, to really put care into everything and to be very vigilant.

(Job Coach 2)

The second theme regards what the training lacked in terms of remaining issues in the team’s practice that GPM cannot address. The codes all relate to challenges that job coaches face and are difficult to address, although the participants did not all mention each of them: patients’ recurring reluctance to respond to the need for the job coaches to contact patients’ former employers to gain a better understanding of their situation, the lack of motivation, patients’ professional plan and work capacity, the impossibility to adapt fully IPS to the BPD population, and the limitations of the labour market, notably the rather protective quality of the Swiss one compared to the United States where the IPS model originated. The following participant notes the difficulty of patients’ refusal to provide access to their former employer’s contact information as a persistent impediment to an optimal intervention that is addressed by neither IPS nor GPM.

The barrier also that I see maybe—it’s true that it’s valuable if you can contact former employers. And in case they refuse, I think it’s complicated, and especially in the case of a PD, I think you have to know a bit how…. Okay, maybe relationally it’s complicated but what was complicated, how did it happen…?

(Job Coach 5)

Several conditions were mentioned as being required to apply the GPM principles. This constitutes the third theme, which is driven by the idea that close collaboration with the patient’s care network, a recognised BPD diagnosis, discussion times for the team (informally within intervisions and supervisions), and the service’s financial resources are needed for optimal implementation. This is reflected in a participant’s discourse praising the benefits of intervisions for their practice as well as the importance of involving patients’ care networks for improving the intervention effectiveness.

Intervision remains the best form of continued training; It’s the culture of exchange. In our profession, without exchange, you become poorer. That’s clear. In addition, with this type of people or profiles […], it can call into question your professional identity, your doubts. It’s true that sometimes you waver too, you see, so there’s always this work of distancing to be done—relationally, being too close, too far away, and so on—so the third party of the colleagues, or of the group, regulates, and then brings some material to the prevention of oneself, to save one’s energy. It’s important and it seems essential to me.

(Job Coach 4)

The last theme gathers the negative aspects of GPM the job coaches listed. It is the less representative theme because it is composed of only four codes from which none are general to the whole sample. GPM incompatibility with the job coach role was noted. They feared being put in the place of a therapist rather than a job specialist, notably with psychoeducation. In addition, three participants raised the negative vision of this implementation as extra work, and the difficulty to automatise the use of the tools in their practice. Compatibility between GPM and some IPS principles—time-unlimited support and priority to patients’ preference—was also questioned. In the following quotation, where a participant was asked whether GPM and IPS were compatible, the participant answered that GPM was going against the IPS principle of time-unlimited support through its incitement to frame an intervention and assess its usefulness for continuing, notably depending on the patient’s engagement. However, this IPS infringement seemed helpful when working with people with BPD.

Clearly, if you take the die-hard principles of IPS, well, no. Because if you really take IPS at its core, it’s, "we’ll support you until there’s no more need." Now, with these people, it’s an unlimited need […] So it could help us frame, yeah, all of that, with empowerment and so on.

(Job Coach 1)

*Part 2*: *Service implementation outcomes 9 months after the GPM training*

Job coaches’ perspectives about the implementation 9 months after their GPM training appear in Table 2. The main themes are the same as the ones present in the focus group, with an additional one regarding progress margin in the implementation, which could obviously not emerge right after the training. All themes of Part 2 were general except the last one, which was typical.

**Table 2:** Analysis of individual placement and support job coaches’ discourse regarding the integration of good psychiatric management nine months after training.

Themes	Codes
Positive impact of the GPM training in the context of IPS (*N* = 6)	Principles’ adoption (*N* = 6) Promotion of comfort and skills (*N* = 6) Supporting supervisions (*N* = 6) Usefulness for other disorders (*N* = 6) Generalisable training in psychiatry (*N* = 6) Mostly useful for people with borderline personality disorder (*N* = 6) Compatibility with RESSORT (*N* = 5) Serenity in the patient’s care (*N* = 5) Personal comfort beneficial for the patient (*N* = 5) Ease of implementation (*N* = 5) Usefulness of all GPM aspects (*N* = 5) Compatibility with IPS (*N* = 4) Appropriate training content (*N* = 4) Direct application after training (*N* = 4) Better understanding of the disorder (*N* = 3) Compatibility with patients (*N* = 3) Ability to follow individual’s preferences while still providing structure (*N* = 3) No change required in the training (*N* = 2) Appropriation of tools (*N* = 2)
Lacks in the training (*N* = 6)	Inevitable persistence of certain difficulties (*N* = 6) Existence of complementary tools (*N* = 3)
Conditions to apply GPM (*N* = 6)	Regular theoretical reminders necessary (*N* = 5) Exchanges between professionals necessary (*N* = 5) Borderline personality disorder diagnosis necessary (*N* = 4) Practical application necessary (*N* = 4)
Negative aspects of the GPM training in the context of IPS (*N* = 6)	Incompatibility with certain IPS principles (*N* = 5) Limitations to offer psychoeducation in IPS (*N* = 4)
Progress margin for the implementation (*N* = 5)	Room for improvement in good practices (*N* = 5) Ideas for improving the training format (*N* = 5) Ideas for improving the supervisions format (*N* = 4)

*Note*. IPS = Individual Placement and Support; GPM = Good Psychiatric Management.

The richer theme of this part is the positive effect GPM has on the team. It shows how most GPM principles were adopted in the IPS programme, how it helped the job coaches feel more comfortable in their practice, and how this positively affects the patients. They also found GPM compatible with their initial practice and would recommend the generalisation of the training in psychiatry. It is possible to see this, for example, in a participant’s discourse.

Typically, in this situation, everything went like clockwork, even though he was the most temperamental patient I had. So yes, I think being clear, naming things, and being prepared, with an eye steeped in the training we’ve had, helps a lot, really a lot. Because before, I know there would have been a huge reaction, whereas by doing that, it allows you to anticipate, it allows you to prevent, it allows you to communicate better; There’s no ambiguity. So, it sounds silly like that, but I wouldn’t have done it before. […] Because it’s about saying things, being as clear as possible. And that helps enormously in this type of care because there is no room for interpretation, there is no room for triangulation, for splitting. In any case, it avoids this kind of thing. So, I find it more comfortable since the training.

(Job Coach 6)

The second theme is about what the training lacks in the sense that GPM cannot overcome all aspects of what can arise in IPS practice. Patients will always face difficulties when a job coach follows them. Additionally, GPM alone is not the only tool that allows job coaches to feel more comfortable and it is not meant to replace all types of treatment modalities. Notably, one participant described this.

Good treatment requires patient adherence. And if you don’t have that, even the best PD psychiatrist who has a good success rate with those who adhere, well, it’s not going to work. That’s why I say that the training, the content, is good; I found it very good. At the same time, there are other independent variables that can play a role.

(Job Coach 1)

The third theme aroused the idea that GPM was applicable only under certain conditions, including the necessity of theoretical reminders, practical application of this theory, a space to discuss cases, and the presence of an official BPD diagnosis. One participant explained how important regular reminders about good practices were important for their work with BPD patients.

I find that, unfortunately, with borderline patients, even if we think we know, we always need a reminder to be able to cope during the intervention, to be able to maintain the framework, [and] to meet the patients’ needs.

(Job Coach 3)

Yet, the team shared some negative aspects of the training, notably that it was going against a few IPS principles, and that psychoeducation, being a key element of GPM, was not the job coaches’ role. One participant explained why the IPS principle of competitive employment as a first goal was not always advised for BPD patients.

Normally, that’s the request. In reality, given that there are things that have been experienced as a failure, that have been destabilising, emotionally hurtful for these people, sometimes, lowering the professional stakes and tending towards experiences that are going to be more reassuring, containing, perhaps not in the competitive work market, will have, in my opinion, a constructive impact on them for the future […]. In fact, what we are aiming for is duration, sustainability; so I allow myself to readjust things so that a foundation of trust is established for the person and for the intervention. That’s the only way to move the relationship forward.

(Job Coach 2)

The last theme relates to the progress margin for a more successful implementation. It includes the improvements the job coaches can make towards better practices—the principles they need to integrate further—, but it also includes suggestions to improve the training and associated supervisions. One participant confessed she still had difficulties not overreacting to what the patient provoked, which is a key principle of the GPM approach. However, we still saw her motivation to improve this point.

I’m still overreactive. That’s the whole point of continuing to train not to be. Or less, at least.

(Job Coach 2)

## Discussion

To the best of our knowledge, this is the first qualitative study investigating the use of GPM in an IPS context with service providers at two time points. The implementation of a training for good practices for BPD in a supported employment setting was assessed soon after the training and 9 months later, and the main attitude seemed positive. Quantitatively, there were more themes and codes on the positive side than on the negative one, and participants less often shared the latter.

Feasibility or practicability can be considered reached because, soon after the training, job coaches described GPM as compatible with their practice, and that several theoretical points were elucidations of the way they were already working. After a few months, they described an ease in implementing GPM principles. However, according to them, this application was possible only under some circumstances, such as a well-functioning care network collaboration and the presence of a BPD diagnosis, which are largely encouraged within GPM theory. These requirements do not only fall under the coaches’ responsibility, but the latter do have some latitude to bring progress, such as involving the first-line therapist more, as it should be a condition for IPS participation. Some coaches in this study even suggested imposing collaboration with patients’ psychotherapists.

Acceptability, which the expression of the job coaches’ satisfaction can assess, was high. Directly after the training, they found GPM theory interesting, and after a few months, they felt more comfortable and skilled. They were also positive about the method of implementation: They notably appreciated assisting group supervisions even though this could have been seen as an extra burden. Nevertheless, they sensed potential issues soon after the training: the additional workload the integration induced, as well as the expected difficulty in automatising the use of new tools. However, these matters are not negative points about the combination of GPM and IPS per se, and they were no longer mentioned after a few months of practice.

Regarding appropriateness or suitability, soon after the training—this opinion persisted after a few months of practice—the job coaches saw a use for GPM. They recognised its advantages and benefits in their work with BPD patients. They found it adapted to other patients too. In that sense, they suggested systematising the GPM practice in other psychiatric contexts. No detrimental aspect was mentioned. Compatibility with their clinical model of practice was recognised, except regarding some IPS principles, which they felt like they were violating by following the GPM approach. In fact, it depends on IPS theory’s interpretation: IPS and GPM principles can actually coexist. IPS values patients’ professional project preferences while BPD patients tend to come regularly with new goals—due to BPD patients’ self-image instability notably marked by shifts in vocational aspiration [13]—hampering the likelihood of any project to materialise. However, IPS does not state that job coaches should follow patients through ever-changing goals. With the GPM principles in mind, it is possible to support the patient according to their preferences with enough frame to keep this goal in mind and to avoid repetitive deviations. This is precisely what half the job coaches raised during the interviews. Similarly, there was a seeming contradiction between the IPS principle of time-unlimited support and GPM-framed intervention. It is again the interpretation of IPS principles that causes confusion because job coaches could theoretically continue supporting patients as long as it makes sense for them. However, IPS does not affirm that the intervention should continue when there is no more sense to it. Instead, job coaches ought to use their common sense and end the intervention if it is not helping. GPM specifies principles that already exist. Job coaches also noticed an incompatibility of GPM with some features of their professional function. This is exactly why GPM theory argues for multimodality of treatment, so that each practitioner avoids going beyond their role. Furthermore, psychoeducation, for example, was not always mentioned in its exact definition during the interviews. It is indeed not the job coach’s role to educate patients fully about their disorder, but they can talk about BPD patients’ functioning related to work, for instance. Moreover, all job coaches mentioned offering psychoeducation as one of the adopted principles, which shows they all practiced it to some extent.

Adoption, or uptake of the GPM assets into IPS practice, occurred according to the job coaches. After 9 months of training, they reported applying the principles, feeling more comfortable and skilled, and having integrated the tools that spread through their professional style. Yet, they noted that an improvement margin still existed to be able to affirm truly that they fully practiced GPM, which was not surprising at this stage.

Fidelity was defined as the adherence to GPM. Again, after a few months of training, job coaches revealed applying most principles. However, job coaches stated several issues: remaining difficulty in setting goals with unstable patients and the need for a functioning collaboration with the patient’s network, which depends not only on them but on mutual will. In addition, as mentioned, it seemed to them that it was impossible to follow GPM principles and some IPS principles simultaneously. However, IPS, being a flexible model, is subject to interpretation. With a critical view, it is possible to be faithful to both models simultaneously. In addition, IPS in its classical form did not seem to be adapted to the BPD population [12]; Therefore, it is expected to deviate from the conventional principles to achieve better effectiveness.

The job coaches mentioned some of what the training lacked, including issues that were indeed not addressed by GPM, which was not originally designed for supported employment and cannot include every specific matter of each modality of treatment involved in a patient’s path towards recovery. Nevertheless, we believe that some of these concerns are subject to improvement, and with the right tools, the job coaches could address them, such as the use of motivation-based approaches. Larson [27] suggested coupling supported employment with motivational interviewing. This would require resources and more evidence but is an interesting avenue. Job coaches could also focus on defining an intervention goal until they become more comfortable with this task. This could fall into other aspects that coaches mentioned as margin of progress for them. Conversely, some of the job coaches’ concerns are rather independent of their tasks, such as navigating low work capacities or the constraints of the Swiss economy that leaves little opportunity for less efficient workers. Some level of difficulties is also unavoidable: Patients’ health still depends more on psychotherapeutic work than on the job coaches who are not meant to be in the first line or in charge of treatment. Furthermore, even though GPM is applicable and good enough for most patients, some of them will still need BPD-specialised therapies to evolve positively [15].

To support the present results, qualitative and quantitative studies are also ongoing with BPD patients participating in IPS at RESSORT. We aim at analysing if the attitude of trained job coaches translates into patients’ satisfaction and better success in terms of professional reintegration. The following step would then be to compare IPS effectiveness with and without GPM-trained job coaches in a RCT, and then work on the implementation methodology to generalise the use of GPM in other teams.

A limitation of the study is that almost all participants were women. However, this is representative of RESSORT team and social professionals in general [28]. Also, the sample size for both the focus group and the interviews was limited, and despite our assessment indicating saturation, this concept continues to be a controversial issue within the qualitative research literature [24]. One potential bias is that the same person gave the training who also conducted the focus group and interviews with job coaches, which could have induced a will to please the trainer in giving positive feedbacks. Aware of this possible issue, the authors adopted a reflexive posture, and the analyses were discussed with several team members. Furthermore, no objective data was collected from job coaches to assess adherence to GPM as in the study by Kolla et al [29], attitude as did Keuroghlian et al [20] and Masland et al [21], or competence, for example. Finally, this study was led in Vaud, Switzerland, and the results may not generalise to all IPS teams.

## Conclusion

Through the IPS team, this study shows the feasibility, acceptability, appropriateness, adoption, and fidelity in implementing GPM practices in the IPS model. Indeed, job coaches were mostly positive about this feature. They all demonstrated their interest and the added value of such an intervention, which constitutes a reinterpretation of IPS principles rather than a real alteration of the model, notably regarding the focus on patients’ preferences, support time limitation, and psychoeducation about work limitations. Improvement ideas include the method of implementation as the integration of GPM in IPS does not seem to contain any theoretical barriers. Among other elements, it would be beneficial to encourage more care network collaboration, which are both significant features of IPS and GPM approaches, emphasise the ways to combine IPS and GPM during the training, increase the intervention frame’s clarity with patients—which could all be addressed during supervisions—, reinforce supported employment teams’ fidelity to the IPS model, which is a major component for supported employment success [30], or suggest the use of motivation-based approaches [27] as an extra feature. However, these results are promising and should be tested further in the hope to increase IPS effectiveness for BPD patients.
